# Correction to: JCcirc: circRNA full-length sequence assembly through integrated junction contigs

**DOI:** 10.1093/bib/bbae062

**Published:** 2024-02-23

This is a correction to: Jingjing Zhang, Huiling Zhang, Zhen Ju, Yin Peng, Yi Pan, Wenhui Xi, Yanjie Wei, JCcirc: circRNA full-length sequence assembly through integrated junction contigs, *Briefings in Bioinformatics*, Volume 24, Issue 6, November 2023, bbad363, https://doi.org/10.1093/bib/bbad363.

In the originally published version of this manuscript, there were errors in the order and content of the funding statement. This originally read:

Youth Innovation Promotion Association (Y2021101), CAS to Y.W. Partly supported by the Key Research and Development Project of Guangdong Province (grant no. 2021B0101310002), National Key Research and Development Program of China (grant no. 2021YFF1200100) Strategic Priority CAS Project XDB38050100, National Science Foundation of China (grant no. 62272449) and the Shenzhen Basic Research Fund (grant nos. RCYX20200714114734194, KQTD20200820113106007 and ZDSYS20220422103800001).

It has now been corrected to:

This work is partly supported by the Key Research and Development Project of Guangdong Province under grant no. 2021B0101310002, the National Science Foundation of China under grant no. 62272449, the Strategic Priority CAS Project under grant no. XDB38050100, the National Key Research and Development Program of China under grant no. 2021YFF1200104, the Shenzhen Basic Research Fund under grant nos. RCYX20200714114734194, KQTD20200820113106007 and ZDSYS20220422103800001, and by the Youth Innovation Promotion Association (Y2021101), CAS to Yanjie Wei.

